# New open drug activity data at EBI

**DOI:** 10.1186/1752-153X-3-S1-O3

**Published:** 2009-06-05

**Authors:** C Steinbeck, B Al-Lazikani, H Hermjakob, J Overington, J Thornton

**Affiliations:** European Bioinformatics Institute, Hinxton Cambridge, CB10 1SD UK

**Keywords:** Public Domain, Team Leader, Target Class, Chemistry Literature, Informatics System

The Wellcome Trust has awarded £4.7 million to the European Bioinformatics Institute (EBI) to support the transfer of a large collection of information on the properties and activities of drugs and a large set of drug-like small molecules from BioFocus DPI, part of the publicly listed company Galapagos to the public domain.

The databases will be incorporated into EBI's collection of open-access data resources for biomedical research and will be maintained by a newly established team at the EBI. These data lie at the heart of translating information from the human genome into successful new drugs in the clinic.

The databases to be brought into the public domain include DrugStore™ (database of known drugs), StARLITe™ (database of known compounds and their effects), Strudle™ (binding site drugability), and Kinase SARfari™ and GPCR SARfari™ (informatics systems for the most widely used target classes in drug discovery). The main database, StARLITe, on Drug-Target interactions alone has hundreds of thousands of interaction data points manually curated from the medicinal chemistry literature.

A new team leader will be appointed to support the new resource and my group will provide the chemoinformatics expertise to move the underlying analysis software into the open source world. The Chemistry Development Kit (CDK) will play a major role both in freeing the QSAR code as well as in providing (sub-) structure and similarity searching to the database.

The transfer will empower academia to participate in the first stages of drug discovery for all therapeutic areas, including major diseases of the developing world. In future it could also result in improved prediction of drug side-effects and spark all kinds of new academic research directions.

This talk will outline the resources to be brought into the public domain, discuss the integration into EBI resources and give a view on our future plans of developments.

